# Phenol Profiling and Nutraceutical Potential of *Lycium* spp. Leaf Extracts Obtained with Ultrasound and Microwave Assisted Techniques

**DOI:** 10.3390/antiox8080260

**Published:** 2019-07-31

**Authors:** Luna Pollini, Rachele Rocchi, Lina Cossignani, Jordi Mañes, Dario Compagnone, Francesca Blasi

**Affiliations:** 1 Department of Pharmaceutical Sciences, Section of Food Science and Nutrition, University of Perugia, via San Costanzo, 06126 Perugia, Italy; 2 Faculty of Bioscience and Technologies for Food, Agriculture and Environment, University of Teramo, via Renato Balzarini 1, 64100 Teramo, Italy; 3 Laboratory of Food Chemistry and Toxicology, Faculty of Pharmacy, University of Valencia, Av. Vicent Andrés Estellés s/n, 46100 Burjassot, Spain

**Keywords:** antioxidant activity, food waste, *Lycium* leaves, phenol profiling, extraction methods

## Abstract

In recent years, agricultural and industrial residues have attracted a lot of interest in the recovery of phytochemicals used in the food, pharmaceutical, and cosmetic industries. In this paper, a study on the recovery of phenol compounds from *Lycium* spp. leaves is presented. Ultrasound-assisted extraction (UAE) and microwave-assisted extraction (MAE) have been used with alcoholic and hydroalcoholic solvents. Methanolic UAE was the most successful technique for extracting phenols from *Lycium* leaves, and we used on leaves from *L. barbarum* and *L. chinense* cultivated in Italy. The extracts were then characterized as regards to the antioxidant properties by in vitro assays and the phenol profiling by a high performance liquid chromatography-diode array detector (HPLC-DAD). Chlorogenic acid and rutin were the main phenol compounds, but considerable differences have been observed between the samples of the two *Lycium* species. For example, cryptochlorogenic acid was found only in *L. barbarum* samples, while quercetin-3-O-rutinoside-7-O-glucoside and quercetin-3-O-sophoroside-7-O-rhamnoside only in *L. chinense* leaves. Finally, multivariate statistical analysis techniques applied to the phenol content allowed us to differentiate samples from different *Lycium* spp. The results of this study confirm that the extraction is a crucial step in the analytical procedure and show that *Lycium* leaves represent an interesting source of antioxidant compounds, with potential use in the nutraceutical field.

## 1. Introduction

The genus *Lycium* belongs to the Solanaceae family and it includes numerous species which grow in arid and semi-arid regions, such as South Africa, America, Australia, and Europe [1]. It has been suggested that the original habitat of *Lycium* spp. was located in the Mediterranean Basin [2].

Products from *Lycium barbarum* and *Lycium chinense* today are considered a “superfood”. *Lycium* species are perennial shrubs or small trees, characterized by fast growth, a root system developed in depth, and good tolerance for drought and cold [3].

Recently, numerous papers reported on the phytochemical composition of goji berries [4,5,6]. Some studies claim that these fruits are rich in bioactives with antioxidant activity, such as carotenoids, flavonoids, and phenolic acids [7,8,9]. In many countries around the world, fruits are widely consumed fresh, dried, and transformed into food (as juice, in wine or tea preparation, in soups, and added to meat and vegetable dishes).

Other parts of the plant (leaves, stems, flowers, and roots) are used as ethno-medicinal food [10,11]. In their origin area, leaves of *L. barbarum* and *L. chinense* are commonly used fresh, cooked, or dried for tea preparation. *L. barbarum* leaves are solitary or fasciculate, lanceolate or long elliptic. *L. barbarum* leaves are narrower than *L. chinense* leaves, which are solitary or in clusters of 2–4 at blade ovate or rhombic [1,12]. However, the phytochemical composition of *L. barbarum* leaves is less studied than *L. chinense* leaves [13].

Recently, it has been reported that *L. barbarum* leaves contain a polysaccharide–protein complex, rich in carbohydrates (including arabinose, galactose, glucose, mannose, rhamnose, ribose, and xylose), uronic acid, and calcium. This complex exhibits interesting health properties, among which are anticoagulant and antiplatelet activities [14]. As regards phenol compounds, the most abundant is rutin (quercetin-3-*O*-rutinoside), but also chlorogenic acid and scopoletin have been reported. Moreover, a significant level of tannins, phenolic acids, and flavonoids, among which are catechin and neohesperidin, was reported [15]. This chemical composition may change according to the type of plant: It was shown that rutin is the major component in wild and cultivated *L. barbarum* leaves, while chlorogenic acids and flavonoid glycosides are found abundantly in cultivated plants and kaempferol-3-*O*-rutinoside is found in wild plants [16].

Terpenoids are the most interesting compounds in *L. chinense* leaves, which also contain steroids, flavonoids, and phenolic acids, among which are rutin, quercetin, kaempferol, chlorogenic, ferulic, and *p*-coumaric acids [13]. Other miscellaneous compounds include free amino acids, such as proline, histidine, alanine, and free sugars, among which are fructose, glucose, sucrose, and maltose. Olatunji et al. investigated the effect of *L. chinense* leaf extracts in rats with diabetic nephropathy and found that leaves are able to manage hyperglycemia and hyperlipidemia and, as a result, they could be used to treat and prevent diabetic nephropathy [17].

In recent years, agricultural and industrial wastes have attracted a lot of interest in the recovery of antioxidant compounds of potential use in food, pharmaceutical, and cosmetic industries. In this regard, recovery of phytochemicals from these products is typically achieved through different extraction techniques [18]. It has been reported that various solid-liquid extraction techniques are widely used for isolating plant antioxidants [19]. Generally, the extraction methods can be split into classical and innovative procedures [18]. The first procedure (i.e., maceration) uses conventional solvents without heat or with thermal treatment to improve the efficiency; these methods are easy to use, but have high-solvent consumption. On the contrary, innovative extraction techniques, among which are supercritical fluids, ultrasounds, and microwaves, allow us to ameliorate the extraction efficiency and/or selectivity by using processing aids/energy inputs [20].

In this research, ultrasound-assisted extraction (UAE) and microwave-assisted extraction (MAE) have been used with the objective to investigate the most effective technique to extract phenol compounds from a *L. barbarum* leaf sample from central Italy. A comparison with the traditional maceration (MAC) technique has been performed. Alcoholic and hydroalcoholic extracts were characterized considering their in vitro antioxidant properties and phenol composition. The profiling of phenol compounds isolated from *Lycium* leaves was studied by a high performance liquid chromatography-diode array detector (HPLC-DAD). The data of phenol contents have been processed by multivariate statistical techniques in order to evaluate the possible discrimination of *L. barbarum* and *L. chinense* samples.

## 2. Materials and Methods

### 2.1. Plant Materials

*L. barbarum* and *L. chinense* fresh leaves were collected in 2017 in different areas of Umbria (central Italy). Damaged leaves were manually discarded. Undamaged intact leaves were dried in a ventilated oven (Binder, Series ED, Tuttlingen, Germany) at 40 °C for 72 h, and in any case until a constant weight was reached. Finally, dried leaves were grounded in a blender and passed through a 250 µm sieve to obtain a fine powder (moisture 10 ± 1%). These samples were stored in amber glass containers away from light and humidity at room temperature, until extraction. One sample of *L. barbarum* leaves was used for the comparison of different extraction methods, while four different *L. barbarum* leaf samples (1B–4B) and *L. chinense* leaf samples (1C–4C) have been analyzed to characterize the phenol fraction and evaluate the possible discrimination of *Lycium* spp.

### 2.2. Reagents

2,2′-azino-bis(3-ethylbenzothiazoline-6-sulphonic acid) diammonium salt (ABTS), 2,2-diphenyl-1-picrylhydrazyl (DPPH radical), Folin and Ciocalteu’s phenol reagent, gallic acid, (±)-6-hydroxy-2,5,7,8-tetramethylchromane-2-carboxylic acid (Trolox), 2,4,6-tripyridyl-s-triazine (TPTZ), chlorogenic acid (≥95%), *p*-cumaric acid (≥98%), ferulic acid (99%), kaempferol-3-*O*-glucoside (≥95%), rutin (≥95%), and tyrosol (98%) were from Sigma–Aldrich (Milan, Italy). Ultrapure water, formic acid, and acetonitrile were Ultra-High Performance Liquid Chromatography-Tandem Mass Spectrometry (UHPLC−MS) grade and were purchased from Carlo Erba Reagents (Milan, Italy). The other solvents were purchased from VWR (Milan, Italy).

### 2.3. Extraction Methods of Goji Leaves

#### 2.3.1. Ultrasound-Assisted Extraction (UAE)

Dried *Lycium* leaf samples (330 mg) were extracted with 20 mL of pure methanol (UAE 1) and methanol/water 50:50 *v/v* (UAE 2) for 30 min at 45 °C using UAE (sonication bath mod. AU-65, ArgoLab, Carpi, Italy). The ultrasonic power was 180 W. The extract was then filtered through paper filter (MN 615, Macherey–Nagel, Düren, Germany), collected in amber glass vials, and kept at −20 °C until further analysis. The extraction was repeated three times.

#### 2.3.2. Microwave-Assisted Extraction (MAE)

Dried *Lycium* leaf samples (330 mg) were extracted with 20 mL of pure methanol (MAE 1) and methanol/water 50:50 *v/v* (MAE 2) for 30 min at 45 °C using a closed vessel system microwave (Model Initiator 2.0, version 2.3, Biotage AB, Uppsala, Sweden) under controlled conditions. The temperature was the preferred controlled variable to avoid degradation of the target compounds and to achieve the maximum efficiency. The other parameters were directly dependent on the temperature, such as the magnetron power (maximum 40 W) and pressure (maximum 5 bar). At the end of the treatment, the vessel used was cooled to room temperature. The extract was filtered through paper filter (MN 615, Macherey–Nagel, Düren, Germany), collected in amber glass vials, and kept at −20 °C until further analysis. The extraction was repeated three times.

#### 2.3.3. Maceration (MAC)

Dried *Lycium* leaf samples (330 mg) were extracted for 4 h min at room temperature while being stirred, using a dynamic maceration with 20 mL of pure methanol (MAC 1) and methanol/water 50:50 *v/v* (MAC 2). The extract was filtered through paper filter (MN 615, Macherey–Nagel, Düren, Germany), collected in amber glass vials, and kept at −20 °C until further analysis. The extraction was repeated three times.

### 2.4. Determination of Total Phenol Content (TPC)

The total phenol content (TPC) was determined spectrophotometrically according to the method of Singleton and Rossi (1965), as modified by Pagano et al. [21]. Folin and Ciocalteu’s phenol reagent was used and the absorbance was measured at 765 nm. The TPC was expressed as mg of gallic acid equivalents (GAE) per gram of dry leaves (mg GAE/g).

### 2.5. In Vitro Antioxidant Activities

#### 2.5.1. Free Radical-Scavenging Activity Using DPPH (DPPH Assay)

DPPH assay was carried out according to the procedure described by Blasi et al. [22]. DPPH methanolic solution was added to each extract. The change in the absorbance of the sample extract was measured at 515 nm after 30 min. The percentage of antioxidant activity (AA%) for each sample was calculated using the following formula:

AA% = (ABSC − ABSS/ABSC) × 100(1)

where ABSC is absorbance of the control solution containing only DPPH and ABSS is absorbance of the DPPH solution containing the sample.

The extract concentration that gave 50% inhibition (IC50) was calculated using the regression equation obtained by plotting the AA% against the extract concentration. IC50 was expressed as mg/mL of the leaf extract.

#### 2.5.2. Free Radical-Scavenging Activity Using ABTS (ABTS Assay)

ABTS assay was performed according to the procedure described by Urbani et al. [23]. A freshly prepared ABTS+ solution was added to the sample extracts and the absorbance was measured at 734 nm after 10 min. The antioxidant capacity of each sample was expressed as mg Trolox equivalents (TE) per gram of dry leaves (mg TE/g).

#### 2.5.3. Ferric Reducing Antioxidant Power (FRAP) Assay

The reducing capacity of the leaf extracts was determined according to the procedure reported by Rocchetti et al. [19]. A ferric reducing antioxidant power (FRAP) reagent was added to the leaf extracts, and then the samples were left in the dark for 30 min. The absorbance of the sample was measured at 593 nm. Aqueous solutions of known Fe^+2^ concentrations (2–5 mM) were used for calibration, and results were expressed as μmol Fe^+2^ per gram of dry leaves (μmol Fe^+2^/g).

### 2.6. HPLC-DAD Analysis of Phenol Compounds

The HPLC analysis of leaf samples was performed according to a previous paper [24]. A pump Thermo Spectraseries, coupled with the Spectra System UV6000LP DAD (Thermo Separation Products, San Jose, CA, USA), was used. The chromatographic separation of polyphenols was carried out with a Hypersil GOLD column (150 × 4.6 mm, 3 μm particle size). The mobile phase solvents were: (A) 0.1% (*v*/*v*) formic acid in water; (B) 0.1% (*v*/*v*) formic acid in acetonitrile. For the analytical separation of the compounds, a gradient profile was employed: Phase B increased from 5% to 20% in 30 min, and then to 95% in 5 min. The mobile phase flow rate was 1 mL/min, while the injection volume was 20 μL.

UV detection was performed, scanning between 280 and 360 nm. The chromatograms were acquired and the data was handled using Xcalibur software version 1.2 (Finnigan Corporation 1998–2000, San Jose, CA, USA).

A standard solution containing phenol compounds (tyrosol, *p*-coumaric acid, ferulic acid, rutin, kaempferol-3-*O*-glucoside, and chlorogenic acid) was used to identify and quantify the analytes. Calibration curves were obtained by three injections of four different concentrations, ranging from 1.5 to 117.2 μg/mL.

### 2.7. UHPLC-MS/MS Analysis of Phenol Compounds

The ultra-high performance liquid chromatography-tandem mass spectrometry (UHPLC-MS/MS) analysis of leaf samples was performed according to Simeoni et al. [25]. An UHPLC system Nexera XR (Shimadzu, Tokyo, Japan) coupled with a 4500 Qtrap mass spectrometer (Sciex, Toronto, ON, Canada) equipped with a heated Electrospray Ionization (ESI) source (V^TM^ source) was used. The ion source parameters were set as follows: Negative ionization mode; ion spray voltage −4.5 kV; air as nebulizer gas at 40 psi, nitrogen as turbo gas at 40 psi; and temperature at 500 °C. The chromatographic separation of polyphenols was carried out with an Excel 2 C18-PFPcolumn (2 μm, 10 cm × 2.1 mm ID-ACE, Aberdeen, UK). The mobile phase was made with the following solvents: (A) Aqueous 0.1% formic acid and (B) acetonitrile. For the analytical separation of the compounds, a gradient profile was employed: 5% phase B was increased up to 100% in 5 min, held for 1 min, and switched back to 5% in 3 min (total time 9 min). The flow rate was 0.3 mL/min. The chromatograms were acquired and the data was handled using MultiQuant 3.0.2 software (AB Sciex, Concord, ON, Canada).

### 2.8. Statistical Analysis

All analytical determinations were performed in triplicate, and the results, expressed as the mean ± standard deviation, were reported on dry leaves. Microsoft Excel 2016 (Microsoft Corporation, Redmond, WA, USA) was used for data analysis. Principal component analysis (PCA) and linear discriminant analysis (LDA) were performed using the Excel-based “XLSTAT” V2006.06 package (Addinsoft, Inc., New York, NY, USA) and the phenol contents obtained by HPLC-DAD analysis were used as variables.

## 3. Results and Discussion

In this research, *Lycium* leaves were extracted by UAE, MAE, and MAC methods, and a comparison among them was carried out. In addition, pure methanol or methanol/water (50:50 *v*/*v*) was comparatively tested to evaluate the impact of the solvent on the recovery of the bioactive phenols from goji leaf powder. Other extraction conditions, such as ratio, temperature, and time, were selected, taking into consideration previous papers [19,22]. Table 1 shows the results of the characterization of the different extracts, regarding the extraction yield, the total phenol content, and the in vitro antioxidant activities.

The percentage of leaf extraction (yield%) was calculated using the following equation:

Yield% (g/100 g) = (W_1_ × 100)/W_2_ (2)

where W_1_ is the weight of the extract residue obtained after solvent removal and W_2_ is the initial weight of leaf powder.

It is possible to observe that the ultrasound technique and the hydroalcoholic solvent gave higher extraction yields. In fact, considering the hydroalcoholic extracts, UAE2 had the highest yield (31.95 %) and MAC2 had the lowest yield (29.82%), while, for methanol extracts, UAE1 gave the highest yield (26.04%) and MAE1 the lowest yield (22.17%).

As regards phenol content, it is possible to observe that the UAE extraction technique with the methanolic solvent was the most effective. In fact, the values were 10.02 and 8.13 mg GAE/g, respectively, for the methanolic (UAE1) and hydro-alcoholic (UAE2) extracts. Additionally, for MAE and MAC techniques, the methanolic extracts showed better results than the hydroalcoholic mixture (6.65 mg GAE/g for MAE1 vs. 5.68 mg GAE/g for MAE2; 9.52 mg GAE/g for MAC1 vs. 5.07 mg GAE/g for MAC2).

Recently, the interest in phenol compounds characterizing goji leaves is due to their health-promoting properties, such as their antioxidant potential [2,10]. Therefore, in this work, the in vitro antioxidant activity of goji leaves was investigated by means of three complementary tests, namely DPPH, ABTS, and FRAP (Table 1). The results obtained for the DPPH radical-scavenging assay, expressed as IC50, ranged from 2.53 of UAE1 to 23.35 mg/mL of MAC2. Higher antiradical activity was found in methanolic extracts (2.52 for UAE1, 3.42 for MAE1 and 3.92 for MAC1), with respect to the hydroalcoholic extracts (9.45 for UAE2, 5.68 for MAE2 and 23.35 for MAC2), independently from the extraction technique. It should be observed that IC50 values are inversely proportional to the antiradical activity, as IC50 represents the sample concentration that gives 50% inhibition of the DPPH radical. Table 1 also shows that ABTS values ranged from 11.68 mg TE/g of MAC 2 to 20.51 mg TE/g of UAE1, with higher values in methanolic than hydroalcoholic extracts, independently from the extraction techniques. The results of the antiradical activity are in agreement with TPC values. In fact, methanolic UAE had the highest antiradical activities and TPC values. Similar findings have been previously reported by Rocchetti et al. [19], who investigated traditional and innovative extraction methods to obtain phenols from *Moringa oleifera* leaves.

Moreover, the FRAP values of the extracts, expressed as μmol Fe^+2^/g, ranged from 55.19 in MAC2 to 140.88 in MAE2. Considering the methanolic extracts, the highest value was obtained for MAE1, while the lowest for UAE1, but generally these values are higher than the values obtained from the hydroalcoholic mixture, with the exception of MAE extracts, where the values are very similar between them (140.74 and 140.88, for MAE1 and MAE2, respectively). An interesting correlation between FRAP and DPPH results (R^2^ = 0.7819) was obtained. These data confirm that the in vitro antioxidant activities were significantly affected by the extraction method.

In this research, HPLC-DAD and UHPLC-MS/MS procedures were performed in order to characterize the phenol components of goji extracts, with the objective to further evaluate the most efficient extraction technique. In particular, the UHPLC-MS/MS technique was carried out for the identification of the analytes, while HPLC-DAD was performed in order to quantify them.

Table 2 shows the parameters obtained from HPLC-DAD analysis of the phenol compounds, used as reference standards

Tyrosol, kaempferol-3-*O*-glucoside, chlorogenic acid, *p*-coumaric acid, ferulic acid, and rutin, with different concentrations (ranging from 1.5 to 117.2 μg/mL), were tested in order to determine the linearity using DAD for this chromatographic method. For each concentration level, injections were performed in triplicate and the average value was used for the external standard calibration curves. For all compounds, the value of R^2^ was good (from 0.9993 of tyrosol to 0.9999 of *p*-coumaric acid). In this research, for intra-day relative standard deviation (RSD), one-day measures of four sample replicates (intra-day precision or repeatability) was considered, whereas for inter-day RSD, samples were analyzed for four consecutive days (inter-day precision or within laboratory reproducibility); it is possible to observe that both intra-day and inter-day precisions were acceptable. The limits of detection (LOD) and limits of quantification (LOQ) were calculated according to the following equations [26]:

LOD = 3.3 × SD/B(3)

LOQ = 10 × SD/B(4)

where SD is the standard deviation of the curve and B is the slope of the curve.

The values of LOD and LOQ (Table 2) show a good sensitivity of the analytical procedure used to determine phenol compounds in *Lycium* leaves.

Table 3 reports the values of the content, expressed as mg/g goji leaves, and the phenol compounds of the different extracts (UAE, MAE, MAC), using the methanol or hydroalcoholic mixture. It can be noted that chlorogenic acid, followed by rutin, is the main phenol compound, with the exception of MAC2, where the extraction was carried out with methanol/water, 50:50 *v*/*v*. In fact, for MAC2, the main compounds were chlorogenic acid (732.71 mg/g) and tyrosol (577.18 mg/g), followed by rutin (452.41 mg/g). The content of chlorogenic acid ranged from 732.71 mg/g of MAC2 to 2991.55 of UAE1. It can be noted that two isomers of chlorogenic acid were also detected (neochlogenic and cryptochlorogenic acids) and their concentrations were determined by the same regression equation of chlorogenic acid.

The obtained results confirm that the extraction method and solvent have a great influence on the chemical composition of the extracts, such as the phenol profile. Our findings are in agreement with the previous investigation of Rocchetti et al. [19], who reported that the extractions carried out with pure methanol were more effective than the hydroalcoholic mixture for phenol compounds recovering from *Moringa oleifera* leaves. Total polyphenol and flavonoid contents of the *Olea europaea* leaf, harvested at the two different stages, were significantly higher in the methanol extract than in less polar fractions [27]. In addition to the analytical procedure, it is well known that the antioxidant capacity of leaf extracts is influenced by several pedoclimatic and agronomic factors, among which are the harvesting period and cultivar [22].

Considering the results reported in Table 1 and Table 3, UAE was more effective than MAE and MAC, and *Lycium* leaves extracted with methanol had more phenol compounds and antioxidant activities than the hydroalcoholic solution extracts. For this reason, in the next step of the research, the UAE methanolic extraction was applied to eight goji leaf samples (four leaf samples of *L. barbarum* and four leaf samples of *L. chinense*) from Umbria (central Italy) region. Table 4 shows the yield, total phenol content, and antioxidant properties of *Lycium* leaf samples.

It can be observed that the yield of extraction ranged from 16.67 to 27.34% for *L. barbarum* samples and from 16.32 to 21.36% for *L. chinense* samples. As regards TPC, the lowest value was obtained for the 3B sample (6.35 mg GAE/g), while the highest was for the 4B sample (19.12 mg GAE/g). The *L. chinense* leaf samples showed more homogenous TPC values, which varied in a small range (from 10.78 mg GAE/g of 4C to 14.37 mg GAE/g of 2C).

The results obtained for *L. chinense* samples showed small ranges of values for DPPH (1.33 mg/mL of 3C–2.21 mg/mL of 1C), FRAP (158.89 μmol Fe^+2^/g of 4C–222.57 μmol Fe^+2^/g of 2C), and ABTS (21.41 mgTE/g of 4C - 26.79 mgTE/g of 2C). On the contrary, greater variability was observed for *L. barbarum* samples, with the 4B sample showing the lowest DPPH value (0.40 mg/mL) and the highest values of FRAP (272.26 μmol Fe^+2^/g) and ABTS (34.27 mg TE/g). Correspondingly, the 4B sample had also the highest value of yield and TPC. On the basis of these results, a correlation study has been carried out considering all the samples. Interestingly, it has been observed that the phenol content correlated well with DPPH (R^2^ = 0.6494), ABTS (R^2^ = 0.9197), and FRAP (R^2^ = 0.9509) values.

In order to obtain the complete profile of phenol compounds in *Lycium* leaf samples, HPLC-DAD and UHPLC-MS/MS were performed. In Table 5, the UV-VIS and mass spectral data used for the identification of the 10 phenol compounds in *Lycium* leaf samples are shown.

Table 6 shows the results of the quantitative analysis of the 10 phenol compounds identified in *L. barbarum* and *L. chinense* leaves by HPLC-MS/MS. Quercetin-3-*O*-rutinoside-7-*O*-glucoside (quercetin-3-*O*-Rut-7-*O*-Glu) and quercetin-3-*O*-sophoroside-7-*O*-rhamnoside (quercetin-3-*O*-Soph-7-*O*-Rha were quantified using the regression equation of rutin, while that of kaempferol-3-*O*-glucoside was used for kaempferol-3-*O*-rutinoside-7-*O*-glucoside (kaempferol-3-*O*-Rut-7-*O*-Glu). As already observed for the *L. barbarum* sample extracted with the different methods, chlorogenic acid and rutin were generally the most represented compounds. These results were also confirmed by Lopatriello et al. for Italian *L. barbarum* leaves and flowers [16]. However, further considerations should be made since more complex profiles have been obtained, showing remarkable differences both for samples of different species and for samples of each species. For example, rutin was not very well represented in samples 1C, 2C, and 4C, while tyrosol was more abundant than rutin in samples 2B, 3B, 2C, and 4C. The main difference between *L. barbarum* and *L. chinense* leaves concerns quercetin-3-O-Rut-7-O-Glu and quercetin-3-O-Soph-7-O-Rha, compounds detected only in *L. chinense* leaves. Moreover, cryptochlorogenic acid was found in all *L. barbarum* samples but in none of the *L. chinense* samples. As regards phenolic acids, ferulic acid was quantified in all *L. chinense* samples (198.96 for 2C–1201.89 for 4C µg/g), while it was detected in small concentrations only in 3B (14.58 µg/g) and 4B (10.53 µg/g). Moreover, *p*-coumaric acid was not detected in *L. chinense* samples, while it was quantified in three *L. barbarum* samples (from 24.55 µg/g in 3B to 585.47 µg/g in 4B).

Mocan et al. studied the phenol compounds in *L. barbarum* and *L. chinense* leaves and reported the presence of three hydroxycinnamic acid derivates, namely ferulic, chrologenic, and *p*-coumaric acids in *L. barbarum* leaves [10]. They found a high amount of chlorogenic acid in the leaves of both *Lycium* spp., with a higher content in *L. chinense* than in *L. barbarum* leaves. Mocan et al. also identified isoquercitrin, rutin, quercitrin, and quercetin. Ferulic acid and kaempferol were detected only in the ethanolic extract of *L. chinense* leaves [10]. Liu et al. studied the compositions of phenolic acids and flavonoids in leaves and stems of the three varieties of *L. chinense* and found that neohesperidin and catechin were the major flavonoids in the leaves, while ferulic, *p*-coumaric, and *p*-hydroxybezoic acid were the major phenolic acids [15]. According to the results obtained in this work, rutin is generally the main flavonoid detected in *Lycium* leaves [2,10].

In order to compare the samples of different varieties (*L. barbarum* or *L. chinense*), a statistic approach was applied to the profiling phenol dataset obtained by HPLC-DAD analysis (data shown in Table 6). Among multivariate statistical data analyses, the most used methods are represented by principal component analysis (PCA) and linear discriminant analysis (LDA). In order to highlight the influence and correlations between variables, phenol results were elaborated by PCA, which reduces the number of potentially correlated variables into a smaller number of uncorrelated factors (principal components). Moreover, LDA is a useful statistical method to examine differences between samples of different groups and to determine the most useful variables for their discrimination. In previous research, lipid analysis and LDA have been applied to animal and vegetable foods for authentication of species or cultivar [28,29,30].

Table 7 shows the eigenvalue, percentage of variance, and cumulative percentage of the principal components, obtained using HPLC-DAD results as variables, relative to the phenol compositions of *Lycium* spp. leaves.

**Figure 1:**
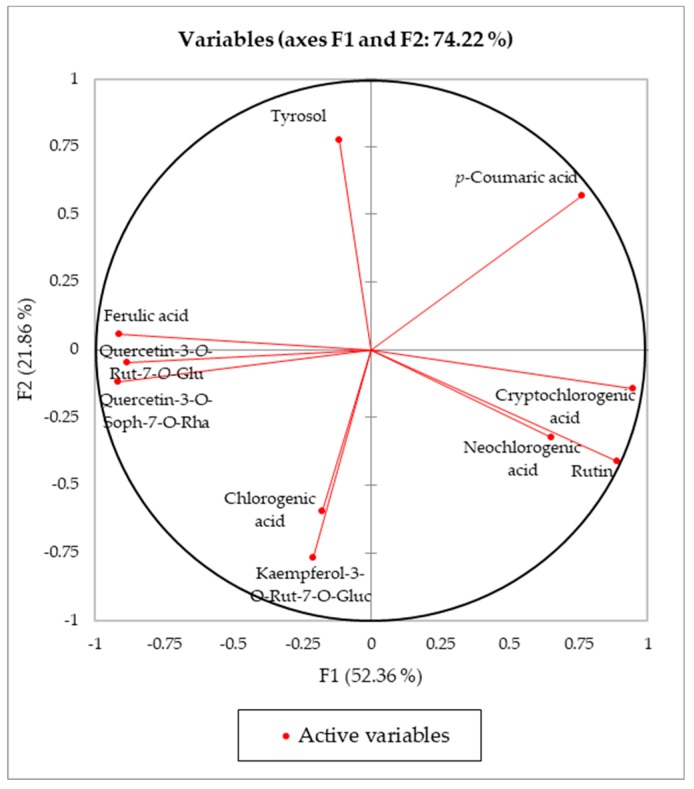
Loading plot for principal components F1 and F2.

The first two components explained 74.2% of the total variance. Figure 1 shows the variable correlation circle (axes F1 and F2); it represents a projection of the initial variables in the factors space.

The correlation circle is useful in interpreting the meaning of the axes. In fact, the horizontal axis is linked with ferulic acid, quercetin-3-*O*-Rut-7-*O*-Glu, quercetin-3-*O*-Soph-7-*O*-Rha, and cryptochlorogenic acid, and the vertical axis with tyrosol, chlorogenic acid, and kaempferol-3-*O*-Rut-7-*O*-Glu. Moreover, it is possible to observe that the vectors of the variables, chlorogenic acid/kaempferol-3-O-Rut-7-O-Glu and neochlorogenic acid/rutin, are grouped, indicating that they are positively correlated.

**Figure 2:**
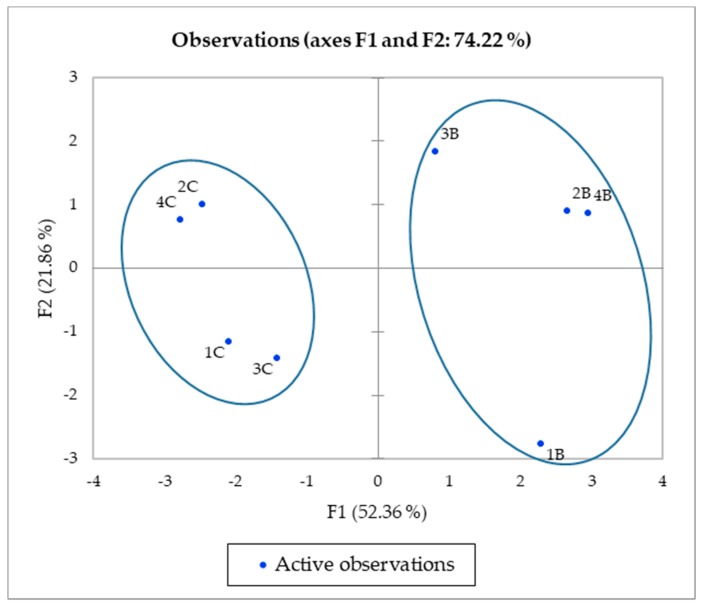
Goji leaf samples on the principal components of the F1/F2 score plot.

In Figure 2, the distribution of goji leaf samples is in the plane defined by the values of the two principal components, according to phenol content. Two main clusters were obtained when considering the different goji leaf extracts. The first cluster, on the right of the plot, was characterized by B1–B4 (methanolic extract of *L. barbarum*), while all the other C1–C4 samples (methanolic extract of *L. chinense*) were included in the second cluster, on the left of the plot.

The multivariate parametric LDA technique was also used in order to classify and discriminate goji leaf samples from *L. barbarum* and *L. chinense* species. The selection of the most significant variables was performed by stepwise analysis. The Wilks’ Lambda test allows us to test if the vectors of the means for the various groups are equal or not. A *p* value lower than 0.001 indicates that the difference between the means vectors of the groups is significant. XLSTAT software selected rutin and cryptochlorogenic acid as variables for *Lycium* samples discrimination.

**Figure 3:**
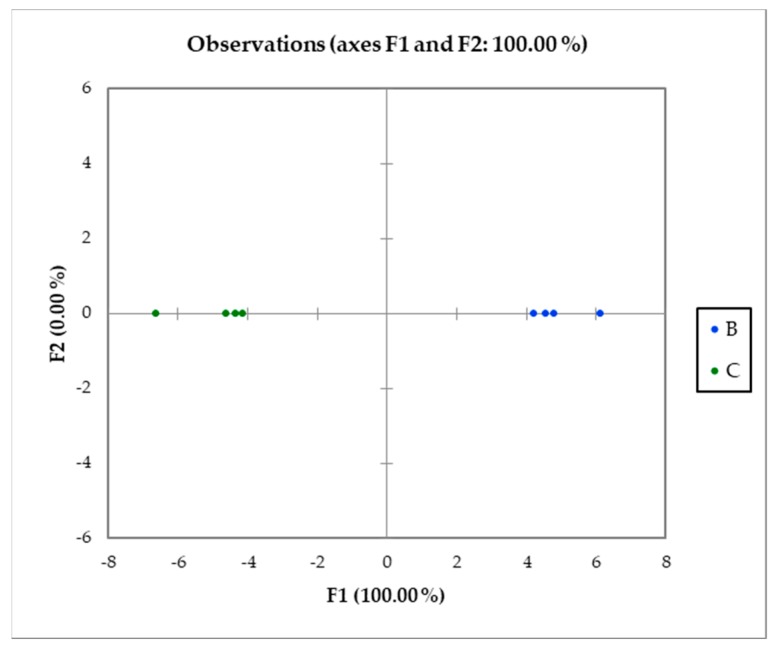
Goji leaf samples on the discriminant function F1 score plot.

Figure 3 shows the plot of the observations on the discriminant function axis, using phenol content data. It allowed us to confirm that the species are very well discriminated on the factor axis extracted from the original explanatory variables. In fact, it was possible to observe that goji samples were well discriminated according to their species (on the right *L. barbarum* samples and on the left *L. chinense* samples). The centroid coordinate on the F1 axis were 4.929 and −4.929 for the B and C group, respectively.

Table 8 summarizes the reclassification of the observations, showing for each observation the factor scores (the coordinate of the observations in the new space), the probability to belong to each group, and the squared Mahalanobis distances to the centroid of each of the classes. Each observation is classified into the group for which the probability of belonging is the greatest. It is possible to observe that all the samples have been correctly reclassified.

The results of classification, reported in Table 9, showed that 100% of original grouped cases were correctly classified. Additionally, to verify the power and the stability of the model, a leave-one-out cross-validation discriminant analysis was performed. From the cross-validation results, it can be observed that 100% of the cross-validated group cases were correctly classified.

The obtained results show that phenol compounds represent useful markers and fingerprinting components for assessing the authenticity of goji leaves.

## 4. Conclusions

In this work, the impact of different extraction technologies (UAE and MAE) was evaluated in terms of recovering and profiling of the phenol compounds from goji leaves by using both alcoholic and hydroalcoholic solvents. The results of the phenol contents and antioxidant capacity showed that methanolic UAE was the most efficient extraction method, compared with the other extraction methods. The obtained results confirm that the extraction technique and solvent have a deep impact on the efficiency of the analytical procedure. These findings are relevant considering that the potential use of ultrasound extraction is promising for the extraction of antioxidants on an industrial scale.

Moreover, in this work, the developed analytical procedure, based on methanolic ultrasound extraction, allowed us to study the phenol profile of leaves from different *Lycium* species. Goji leaf phenol contents, obtained by HPLC-DAD analysis, were processed by PCA and LDA, and the results highlighted the possibility of distinguishing leaves from different *Lycium* spp., despite the limited number of samples. These findings confirm that the phenol profile has a discriminating power for plant-based products and by-products from different species. This approach can be implemented for quality control and authentication of goji leaf-containing foods.

## Figures and Tables

**Table 1:** Yield of extraction, total phenol content (TPC) and in vitro antioxidant activities.

		Yield	TPC	DPPH	ABTS	FRAP
	Extraction Solvent	%	mg GAE/g	IC50 *	mg TE/g	μmol Fe^+2^/g
**UAE 1**	MeOH	26.04 ± 0.13	10.02 ± 0.23	2.53 ± 0.18	20.51 ± 1.62	112.65 ± 2.86
**UAE 2**	MeOH:H_2_O, 50:50	31.95 ± 0.76	8.13 ± 0.06	9.45 ± 0.84	14.65 ± 0.75	92.17 ± 6.64
**MAE 1**	MeOH	22.17 ± 0.79	6.65 ± 0.35	3.42 ± 0.25	17.67 ± 0.81	140.74 ± 0.58
**MAE 2**	MeOH:H_2_O, 50:50	31.48 ± 1.75	5.68 ± 0.38	5.68 ± 0.63	13.14 ± 0.69	140.88 ± 0.61
**MAC 1**	MeOH	24.34 ± 0.86	9.52 ± 0.25	3.92 ± 0.52	14.63 ± 0.92	121.23 ± 3.69
**MAC2**	MeOH:H_2_O, 50:50	29.82 ± 0.62	5.07 ± 0.06	23.35 ± 3.27	11.68 ± 0.86	55.19 ± 0.53

Data are reported as mean ± standard deviation of three independent measurements (*n* = 3) and are expressed on dry weight; * mg/mL.

**Table 2:** Range, regression equation, R^2^, RSD Intradie and Interdie, limits of detection (LOD), and limits of quantification (LOQ) of standard compounds from high performance liquid chromatography-diode array detector (HPLC-DAD) analysis.

Standard	Range	Regression Equation		R^2^	RSD * Intra-Day	RSD * Inter-Day	LOD	LOQ
		Slope	Intercept					
	μg/mL				%	%	μg/mL	μg/mL
**Tyrosol**	7.31–29.24	1.88E + 06	−9.18E + 05	0.9993	1.12	4.98	1.51	4.82
**Kaempferol-3-*O*-Glu** ***^a^***	14.80–59.00	6.06E + 06	7.29E + 06	0.9998	1.13	3.57	0.71	2.10
**Chlorogenic acid**	1.50–117.20	3.38E + 06	−5.20E + 05	0.9996	0.98	3.71	0.68	2.17
***p*-Coumaric acid**	1.78–7.10	1.89E + 07	1.58E + 06	0.9999	0.79	4.80	0.06	0.19
**Ferulic acid**	1.86–7.45	1.68E + 07	1.20E + 06	0.9997	1.72	4.55	0.64	2.05
**Rutin**	11.10–44.30	5.51E + 06	−1.24E + 06	0.9997	1.32	4.13	1.68	5.34

* Relative standard deviation (RSD) (*n* = 4); *^a^* kaempferol-3-*O*-glucoside.

**Table 3:** Content of phenol compounds of *L. barbarum* extracts, obtained with different extraction methods: Ultrasound-assisted extraction (UAE); microwave-assisted extraction (MAE); maceration (MAC).

	UAE 1	UAE 2	MAE 1	MAE 2	MAC 1	MAC 2
**Neochlorogenic acid**	594.04 ± 45.60	412.50 ± 23.11	462.37 ± 25.74	597.91 ± 43.91	450.11 ± 58.40	21.26 ± 2.34
**Tyrosol**	660.61 ± 37.13	640.80 ± 30.73	476.10 ± 34.91	598.19 ± 46.92	501.30 ± 6.40	577.18 ± 40.42
**Chlorogenic acid**	2991.55 ± 46.62	1728.83 ± 110.08	2656.66 ± 150.23	2059.65 ± 104.72	2692.46 ± 166.06	732.71 ± 35.81
**Cryptochlorogenic acid**	375.76 ± 30.55	374.88 ± 4.34	323.91 ± 24.71	346.87 ± 21.94	227.69 ± 28.05	378.29 ± 20.35
***p*-Coumaric acid**	83.81 ± 8.26	81.20 ± 8.42	88.08 ± 7.83	80.20 ± 7.41	33.14 ± 1.55	42.08 ± 3.84
**Rutin**	1678.68 ± 61.74	1197.55 ± 95.89	1328.50 ± 74.91	705.06 ± 34.92	1330.61 ± 184.12	452.41 ± 24.75

Data are reported as the mean ± standard deviation of three independent measurements (*n* = 3) and are expressed on a dry weight basis.

**Table 4:** Yield of extraction, total phenol content (TPC), and in vitro antioxidant activities of *Lycium* leaf samples.

	Yield	TPC	DPPH	ABTS	FRAP
	%	mg GAE/g	IC50 *	mg TE/g	μmol Fe^+2^/g
**1B**	16.67 ± 0.54	14.31 ± 0.12	1.01 ± 0.06	30.18 ± 1.32	194.40 ± 6.85
**2B**	23.84 ± 0.84	8.95 ± 0.08	5.30 ± 0.26	21.91 ± 0.45	138.08 ± 1.15
**3B**	22.78 ± 0.76	6.35 ± 0.14	14.95 ± 1.53	15.46 ± 0.86	76.34 ± 7.92
**4B**	27.34 ± 0.95	19.12 ± 0.26	0.40 ± 0.02	34.27 ± 1.19	272.26 ± 4.94
**1C**	21.12 ± 0.86	12.68 ± 0.51	2.21 ± 0.22	25.62 ± 0.23	165.60 ± 2.36
**2C**	21.36 ± 0.98	14.37 ± 0.24	1.93 ± 0.12	26.79 ± 0.87	222.57 ± 3.82
**3C**	16.32 ± 0.72	13.54 ± 0.18	1.33 ± 0.09	24.23 ± 0.64	210.19 ± 8.38
**4C**	19.07 ± 0.92	10.78 ± 0.23	2.05 ± 0.21	21.41 ± 1.21	158.89 ± 0.75

Data are reported as the mean ± standard deviation of three independent measurements (*n* = 3) and are expressed on a dry weight basis; * mg/mL; B = *L. barbarum* leaf samples; C = *L. chinense* leaf samples.

**Table 5:** UV-VIS and mass spectral data of the identified phenol compounds.

	R_t_ (min)	λmax (nm)	[M+H]^+^	MS Fragments (m/z)
**Neochlorogenic acid**	8.1	296sh; 324	377[M+Na]^+^	191 [M-H-caffeoyl]^−^; 179 [M-H-quinic]^−^; 707 [2M-H]^−^
**Tyrosol**	8.9	231; 275	137	137 [M-H]^−^; 93 [M-H-CO_2_]^−^
**Quercetin-3-*O*-Rut-7-*O*-Glu** ***^a^***	12.3	255; 266sh; 354	773	611 [M-H-glucose]^+^; 465 [M-H-rutinose]^+^;
**Quercetin-3-*O*-Soph-7-*O*-Rha** ***^b^***	12.8	255; 266sh; 354	773	627 [M-H-rhamnose]^+^; 465 [M-H-rhamnose; M-H-sophorose]^+^;
**Kaempferol-3-*O*-Rut-7-*O*-Glu** ***^c^***	13.3	265; 340	757	611 [M-H-glucose]^+^; 449 [M-H-rutinose]^+^
**Chlorogenic acid**	13.8	244; 296sh; 320	355	191 [M-H-caffeoyl]^−^; 179 [M-H-quinic]^−^; 707 [2M-H]^−^
**Cryptochlorogenic acid**	14.3	244; 296sh; 326	377[M+Na]^+^	191 [M-H-caffeoyl]^−^; 179 [M-H-quinic]^−^; 707 [2M-H]^−^
***p*-Coumaric acid**	20.2	312	163	147 [M-H-H_2_O]^−^; 119 [M-H-CO_2_]^−^
**Ferulic acid**	23.7	238; 290sh; 322	193	193 [M-H]^−^; 178 [M-H-CH_3_]^−^
**Rutin**	26.6	256; 266sh; 354	611	303 [M-H-rutinose]^+^; 1243 [2M+Na]^+^

*^a^* quercetin-3-*O*-rutinoside-7-*O*-glucoside, *^b^* quercetin-3-*O*-sophoroside-7-*O*-rhamnoside, *^c^* kaempferol-3-*O*-rutinoside-7-*O*-glucoside.

**Table 6:** Content of phenol compounds in *L. barbarum* and *L. chinense* leaves.

	**1B**	**2B**	**3B**	**4B**
	**µg/g**	**µg/g**	**µg/g**	**µg/g**
**Neochlorogenic acid**	466.43 ± 9.72	8655.31 ± 266.61	324.82 ± 3.11	508.14 ± 8.88
**Tyrosol**	513.51 ± 18.71	1921.88 ± 19.14	1105.65 ± 24.58	716.23 ± 60.40
**Quercetin-3-*O*-Rut-7-*O*-Glu *^a^***	nd	nd	nd	nd
**Quercetin-3-*O*-Soph-7-*O*-Rha** ***^b^***	nd	nd	nd	nd
**Kaempferol-3-*O*-Rut-7-*O*-Glu** ***^c^***	610.30 ± 38.4	108.25 ± 2.66	99.45 ± 8.51	nd
**Chlorogenic acid**	6354.36 ± 204.81	3048.82 ± 13.93	1353.13 ± 12.24	3139.02 ± 132.54
**Cryptochlorogenic acid**	492.43 ± 65.23	230.46 ± 2.35	161.93 ± 1.17	429.92 ± 6.80
***p*-Coumaric acid**	nd	49.49 ± 0.23	24.55 ± 0.27	585.47 ± 8.80
**Ferulic acid**	nd	nd	14.58 ± 0.44	10.53 ± 1.80
**Rutin**	5756.65 ± 340.5	1808.75 ± 19.37	743.50 ± 4.13	5233.17 ± 264.88
	**1C**	**2C**	**3C**	**4C**
	**µg/g**	**µg/g**	**µg/g**	**µg/g**
**Neochlorogenic acid**	439.58 ± 13.80	325.54 ± 10.91	432.58 ± 7.24	423.96 ± 9.50
**Tyrosol**	596.37 ± 29.25	2057.51 ± 30.74	118.40 ± 1.54	1303.28 ± 43.07
**Quercetin-3-*O*-Rut-7-*O*-Glu** ***^a^***	195.39 ± 2.33	939.49 ± 21.03	268.94 ± 11.07	205.60 ± 3.04
**Quercetin-3-*O*-Soph-7-*O*-Rha** ***^b^***	1946.70 ± 38.95	1011.92 ± 27.54	344.02 ± 5.58	1271.42 ± 16.21
**Kaempferol-3-*O*-Rut-7-*O*-Glu** ***^c^***	380.72 ± 3.07	91.77 ± 4.83	170.21 ± 10.04	366.13 ± 10.04
**Chlorogenic acid**	3811.85 ± 41.41	7721.47 ± 130.84	6056.74 ± 149.80	2153.11 ± 187.22
**Cryptochlorogenic acid**	nd	nd	nd	nd
***p*-Coumaric acid**	nd	nd	nd	nd
**Ferulic acid**	670.72 ± 16.53	198.96 ± 5.39	451.57 ± 12.85	1201.89 ± 24.12
**Rutin**	506.21 ± 8.47	432.63 ± 6.40	1029.45 ± 57.52	375.60 ± 14.95

Data are reported as the mean ± standard deviation of three independent measurements (*n* = 3) and are expressed on a dry weight basis; *^a^* quercetin-3-*O*-rutinoside-7-*O*-glucoside, *^b^* quercetin-3-*O*-sophoroside-7-*O*-rhamnoside, and *^c^* kaempferol-3-*O*-rutinoside-7-*O*-glucoside. Not detected (nd).

**Table 7:** Principal component analysis (PCA): Eigenvalue, percentage of variance, and cumulative percentage.

	F1	F2	F3	F4	F5	F6	F7
**Eigenvalue**	5.235536	2.186099	1.160795	0.671911	0.579965	0.115526	0.050168
**Variability %**	52.35536	21.86099	11.60795	6.719115	5.799649	1.155262	0.50168
**Cumulative %**	52.35536	74.21635	85.82429	92.54341	98.34306	99.49832	100

**Table 8:** PCA: Prior and posterior classification, membership probabilities, scores, and squared distances.

Observation	Prior	Posterior	Pr(B)	Pr(C)	F1	D^2^(B)	D^2^(C)
**Obs1**	B	B	1.000	0.000	6.134	4.188	125.126
**Obs2**	B	B	1.000	0.000	4.793	2.181	96.680
**Obs3**	B	B	1.000	0.000	4.565	3.633	93.645
**Obs4**	B	B	1.000	0.000	4.225	3.258	86.562
**Obs5**	C	C	0.000	1.000	−4.626	92.706	1.487
**Obs6**	C	C	0.000	1.000	−4.347	87.470	1.758
**Obs7**	C	C	0.000	1.000	−6.613	134.879	4.499
**Obs8**	C	C	0.000	1.000	−4.131	83.530	2.087

B = *L. barbarum* leaf group; C = *L. chinense* leaf group.

**Table 9:** Linear discriminant analysis (LDA) classification results.

**Classification Results (Training Sample)**				
**From/to**	**B**	**C**	**Total**	**% Correct**
**B**	4	0	4	100.00%
**C**	0	4	4	100.00%
**Total**	4	4	8	100.00%
**Classification Results (Cross-Validation)**				
**From/to**	**B**	**C**	**Total**	**% Correct**
**B**	4	0	4	100.00%
**C**	0	4	4	100.00%
**Total**	4	4	8	100.00%

B = *L. barbarum* leaf group; C = *L. chinense* leaf group.
